# Inter- and Intra-individual Variability in Brain Oscillations During Sports Motor Imagery

**DOI:** 10.3389/fnhum.2020.576241

**Published:** 2020-10-30

**Authors:** Selina C. Wriessnegger, Gernot R. Müller-Putz, Clemens Brunner, Andreea I. Sburlea

**Affiliations:** ^1^Institute of Neural Engineering, Graz University of Technology, Graz, Austria; ^2^BioTechMed-Graz, Graz, Austria; ^3^Institute of Psychology, University of Graz, Graz, Austria

**Keywords:** EEG, ERD/S, motor imagery, variability, inter-individual differences

## Abstract

The aim of this work was to re-evaluate electrophysiological data from a previous study on motor imagery (MI) with a special focus on observed inter- and intra-individual differences. More concretely, we investigated event-related desynchronization/synchronization patterns during sports MI (playing tennis) compared with simple MI (squeezing a ball) and discovered high variability across participants. Thirty healthy volunteers were divided in two groups; the experimental group (EG) performed a physical exercise between two imagery sessions, and the control group (CG) watched a landscape movie without physical activity. We computed inter-individual differences by assessing the dissimilarities among subjects for each group, condition, time period, and frequency band. In the alpha band, we observe some clustering in the ranking of the subjects, therefore showing smaller distances than others. Moreover, in our statistical evaluation, we observed a consistency in ranking across time periods both for the EG and for the CG. For the latter, we also observed similar rankings across conditions. On the contrary, in the beta band, the ranking of the subjects was more similar for the EG across conditions and time periods than for the subjects of the CG. With this study, we would like to draw attention to variability measures instead of primarily focusing on the identification of common patterns across participants, which often do not reflect the whole neurophysiological reality.

## Introduction

Motor imagery (MI) is defined as an internal representation of simple or complex movements in absence of any physical action or any kind of peripheral muscular activity (Jeannerod, 1994; Annett, 1995; Jeannerod and Decety, 1995; Porro et al., 1996). It is well-known that MI improves motor learning comparable with real physical exercises which results in neural and structural changes in the brain (Grèzes and Decety, 2001; Miller et al., 2010; Sharma and Baron, 2013). Furthermore, MI is a common task in brain–computer interface (BCI) research because users often cannot perform an overt motor execution task due to some degree of motor disability (Neuper et al., 2006; Pfurtscheller and Neuper, 2006; Pfurtscheller et al., 2006; Leeb et al., 2007a; Höhne et al., 2014). With the so-called motor-imagery-based BCI, users send mental commands by performing MI tasks, e.g., movement imagination or attempts (Pfurtscheller and Neuper, 2001; Neuper and Pfurtscheller, 2010; Lotte et al., 2013). Even though improved signal processing and classification algorithms are available, a tremendous inter- and intra-subject variability has been observed in terms of performance (Allison and Neuper, 2010; Wolpaw and Wolpaw, 2012; Kübler et al., 2013). Thus, it is indisputable that one of the major aspects contributing to MI–BCI control performance is the individual characteristic and consequently neural pattern of the BCI user (Kübler et al., 2013; Ahn and Jun, 2015).

In the past years, researchers identified different factors like cognitive, attentional, or personal skills which influence BCI performance (Leeb et al., 2007a, b; Blankertz et al., 2010; González-Franco et al., 2011; Kübler et al., 2011; Halder et al., 2013; Kleih and Kübler, 2013; Lotte et al., 2013; Höhne et al., 2014; Schreuder, 2014). The observed large inter-individual variability motivated researchers to investigate important predictors related to a user’s personality and cognitive profile. Jeunet et al. (2016) suggested the following three categories of MI–BCI performance predictors: (1) users’ relationship with the technology, (2) attention, and (3) spatial abilities. The attention-related predictors seem to be particularly relevant. There is large inter-individual variability in the efficiency of neural activity in the attention network accounting for the inter-individual variations in attentional abilities important for BCI control (Petersen and Posner, 2012). Moreover, several other researchers have identified attention-related brain patterns which are important to BCI performance.

For example, Grosse-Wentrup and Schölkopf (2012) found that the variation in gamma power highly correlates with BCI performance, hence being able to predict successful or unsuccessful classification (Grosse-Wentrup et al., 2011; Grosse-Wentrup, 2012; Grosse-Wentrup and Schölkopf, 2012; Schumacher et al., 2015). Others found that the extent of activation of the dorsolateral prefrontal cortex (associated with the executive attention system; Posner and Petersen, 1990) differs between high and low BCI performers (Halder et al., 2011). Finally, Bamdadian et al. (2014) found that frontal theta, occipital alpha, and midline beta power could be other predictors for BCI performance.

Besides attention, there are several other factors that contribute to a high variability in BCI users. For example, Kübler et al. (2011) suggested a model of BCI control that contains four categories: “Individual characteristics,” “Characteristics of the BCI,” “Feedback and Instruction,” and “Application.” Summarizing this classification, it can be distinguished between two fundamental aspects. One aspect is the user’s part and the other one the system’s part. It has been shown that within the same BCI system, some subjects cannot perform successfully (Allison and Neuper, 2010; Blankertz et al., 2010). These results indicate the importance to understand why some individuals perform differently in the same system. Other researchers like Saha and Baumert (2019) reported that neurophysiological processes during MI often vary over time and across subjects (Meyer et al., 2013; Saha et al., 2017). Because the motor learning process differs across individuals and consequently cortical activity varies among subjects during MI, its utility for BCI applications is largely restricted. Hence, it is very important to more closely investigate inter- and intra-subject variability during MI to find further predictors of inter-individual differences that can improve future MI-based BCI systems.

In a former study, we investigated MI of playing tennis, resulting in different mu rhythm patterns of activation on the basis of individual expertise for the specific task. For instance, experienced tennis players showed a more focal event-related desynchronization (ERD) pattern over sensorimotor regions surrounded by ERS with respect to non-experts (Wriessnegger et al., 2018). Surprisingly, our data clearly show high inter- and intra-individual differences in event-related desynchronization/synchronization (ERD/S) patterns in all tasks and groups reflected in the time–frequency visualization of ERD/S patterns in the alpha band for the same tasks. For example, while one person showed increased ERD during MI of tennis, another one showed increased ERS for the same task. Consequently, an overall analysis of the grand average activity during tennis MI was quite problematic. Such high inter- and intra-subject variability of mu rhythms during MI tasks was also reported by other studies (Doppelmayr et al., 1998; Pineda, 2005; Pfurtscheller and Neuper, 2006; Pfurtscheller et al., 2006; Halme and Parkkonen, 2018; Corsi et al., 2019). For example, Daeglau et al. (2020) attributed the inter-individual differences in MI induced ERD to the experimental setup they used. Concretely, they assumed that task and experimental setup can affect the interplay of motor execution and MI for each individual differently. Others discussed inter-subject variability in the alpha frequency in relation with age and genetic factors, supported by twin studies (Smit et al., 2012; Bodenmann et al., 2009). But also, task demands and cognitive factors like working memory performance influence the alpha peak frequency (Klimesch, 1999). In addition, intra-subject variability in alpha peak frequency has been observed reflecting different alpha networks being activated dependent on task demands (Klimesch, 1999). Following this, alpha frequency can be interpreted as “trait” variable on the one side and “state” variable on the other side. While the former might explain differences in overall cognitive performance among subjects, the latter could explain the observed intra-subject variability. Moreover, this variability might reflect any fluctuations in real-time performance.

These results and the high inter-individual differences in ERD/S patterns elicited in our previous study (Wriessnegger et al., 2018) motivated us to investigate more closely the variability among and between subjects of this dataset. The individual activation patterns during MI are largely neglected in most of the studies which primarily focused on the identification of common patterns across participants.

## Materials and Methods

### Participants

Thirty healthy right-handed students participated in the study. All reported normal or corrected to normal vision and none of them had a history of psychiatric or neurological disorders. Participants were matched with regard to sex and age, and they were randomly assigned to the control group (CG) (*N* = 15; mean age: 24.9; range: 20–30 years; 7 women and 8 men) or to the experimental group (EG) (*N* = 15; mean age: 24.8; range: 20–28 years; 7 women and 8 men). The participants were all naive regarding MI, 70% of them regularly perform different kinds of sports and only five play tennis. The original study was approved by the local ethics committee (Medical University of Graz) and is in accordance with the ethical standards of the Declaration of Helsinki. After detailed written and oral instruction participants gave informed written consent to participate in the study. They received financial compensation (€7.50/hour) for their participation.

### Experimental Design

The experimental procedure encompassed a pre-measurement, the execution or relaxing intervention, and a post-measurement. During the pre-measurement, participants from both groups performed the MI task according to the written instructions while simultaneously their EEG was recorded. Whenever the letter “T” appeared on the screen in front of them, participants had to imagine playing tennis for 6 s repetitively. The concrete instruction was to imagine a repetitive right forehand movement of returning balls from a first-person perspective. If the letter “H” appeared on the computer screen, the task was to imagine squeezing a ball for 6 s with the right hand. Participants had to imagine each type of MI 15 times per run in pseudo-randomized order. The whole experiment consisted of four runs with 60 trials of squeezing a ball and other 60 trials of playing tennis.

During the intervention phase, participants from the EG played virtual tennis via motion control (Kinect) and squeezed a real ball for 5 min each. In this phase, no EEG was recorded. The described execution interventions were performed in randomized order within the EG. In the control group (CG), participants performed no physical exercise, instead they watched a landscape movie for 10 min.

In the last session, after the intervention phase, participants of the EG and the CG performed the same MI (playing tennis and squeezing a ball) tasks like in the first session while their EEG was recorded. A trial consisted of a fixation cross (4 s), the imagery phase (6 s), and a pause (4 s), which leads to a total trail time of 14 s. In one run, 30 trials (15 per MI task) in total are performed, with four runs in the pre-recording and 4 runs in the post-recording phase. Each participant performed eight runs with 240 trials in total. For a more detailed description of the experimental setup, please see Wriessnegger et al. (2018).

### EEG Preprocessing and ERD/ERS Analysis

**FIGURE 1:**
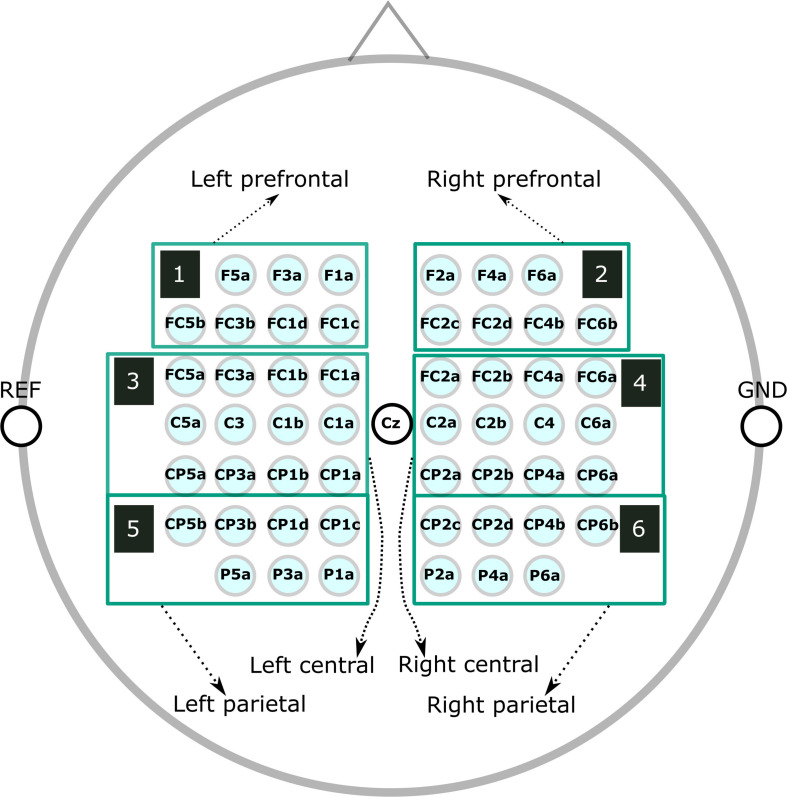
Layout of the EEG channels and description of the ROIs.

The raw EEG data, taken from the original study (Wriessnegger et al., 2018), was down-sampled to 250 Hz and re-referenced to channel Cz. We manually inspected the continuous EEG signals and marked segments containing artifacts, which we discarded in all subsequent analyses. Next, we used non-causal FIR bandpass filters to extract time signals in the bands 8–13 Hz (alpha band) and 16–24 Hz (beta band). We considered segments from −3.5 to 3.5 s relative to each cue for our ERD/ERS calculation, where the baseline and activation intervals ranged from −3.5 to 0.5 s and 0.5 to 3.5 s, respectively. Finally, we averaged groups of channels into the following six regions of interest (ROIs) (Figure 1): prefrontal left (F5a, F3a, F1a, FC5b, FC3b, FC1d, and FC1c), prefrontal right (F2a, F4a, F6a, FC2c, FC2d, FC4b, and FC6b), central left (FC5a, FC3a, FC1b, FC1a, C5a, C3, C1b,C1a, CP5a, CP3a, CP1b, and CP1a), central right (FC2a, FC2b, FC4a, FC6a, C2a, C2b, C4, C6a, CP2a, CP2b, CP4a, and CP6a), parietal left (CP5b, CP3b, CP1d, CP1c, P5a, P3a, and P1a), and parietal right (CP2c, CP2d, CP4b, CP6b, P2a, P4a, and P6a). We computed time/frequency ERD/S maps similar to the procedure used to calculate ERD/S values.

### Calculating Intra- and Inter-individual Differences

For every subject from the two groups (experimental and control), each condition (hand and tennis), time period (pre- and post-intervention), and frequency band (alpha and beta), we averaged the time–frequency patterns during the task (0.5 to 3.5 s) of single channels within each of the six ROIs. Then, we computed the ERD/S patterns and investigated their distribution among subjects during pre- and post-intervention time periods.

Next, we concatenated the average ERD/S values of the six ROIs for alpha and for beta frequency bands for all the subjects, and assessed the dissimilarity between these patterns by means of a pairwise distance computed as 1 - *rho*, where *rho* is the Spearman correlation. We ranked and scaled the distances between 0 and 1, and computed the distance matrix between pairs of conditions. We computed inter-individual differences by assessing the dissimilarities among subjects for each group, condition, time period, and frequency band (Figures 4A,B).

To visualize the distances between different subjects in several conditions, we used multidimensional scaling (MDS) (Kruskal and Wish, 1978). MDS is a general dimensionality reduction method that projects entities in a low-dimensional space, such that their distances reflect their similarities. Specifically, similar entries will be located closer to one another, while dissimilar ones will be farther apart. For MDS visualization as a 2D plot that reflects the distribution of the subjects in terms of their ranking, we performed non-metric MDS for two dimensions with the squared stress criterion.

Next, we investigated the variability of these pairwise distances among subjects in each of the two groups (experimental and control) among conditions, time periods, and frequency bands using Kendall’s tau b and reporting their associated *p*-values. We corrected for multiple comparisons using the Bonferroni–Holm correction.

## Results

### Subject-Specific ERD/S Patterns

**FIGURE 2:**
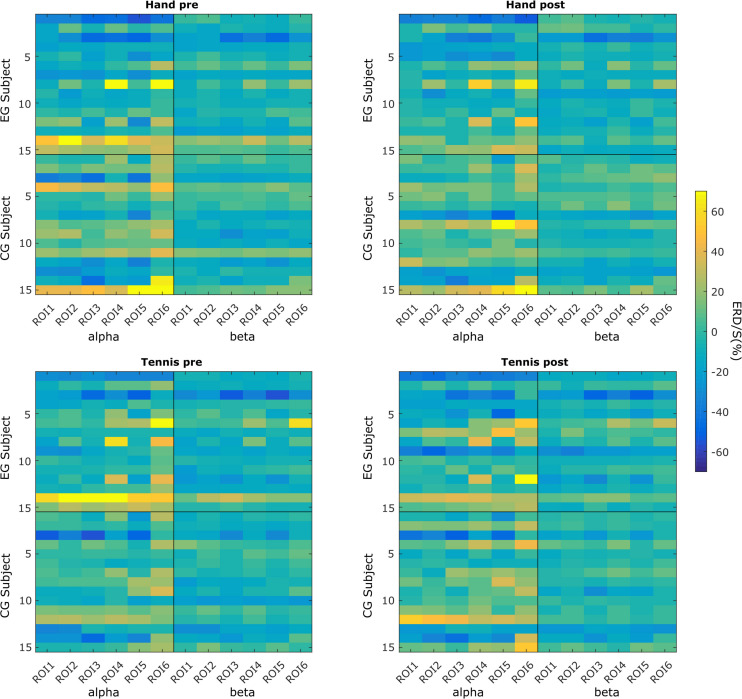
Subject-specific ERD/S magnitudes in each of the six ROIs in the hand and tennis conditions (top and bottom panels), during the pre- and post-intervention time periods (left and right panels) within the alpha and beta frequency bands.

Figure 2 shows subject-specific ERD/S values averaged over the channels of each ROI. In every subplot, the vertical black line separates the values for alpha and beta frequency bands, whereas the horizontal black line separates the two groups of subjects: experimental and control groups. Finally, the top row shows all these patterns for the hand condition: on the left side during the pre-intervention time period and on the right side during the post-intervention time period. Similarly, the bottom row shows these patterns for the tennis condition.

We can observe that for some subjects, the within-subject ERD/S values remain similar throughout the conditions and time points. However, across subjects, these values are very different. For example, subject EG3 shows a strong negative ERD/S value at the level of ROI3 and ROI5 in both hand and tennis pre-conditions for both alpha and beta frequencies. These values slightly increase but remain negative for all the conditions and frequencies in the post-intervention period. However, as another example, subject EG14 presents positive ERD/S values in all conditions. Similar observations can be found in the control group (e.g., CG3 and CG12). We also found that the ERD/S values for the beta frequency show less variability among subjects for either group compared with the alpha frequency band.

**FIGURE 3:**
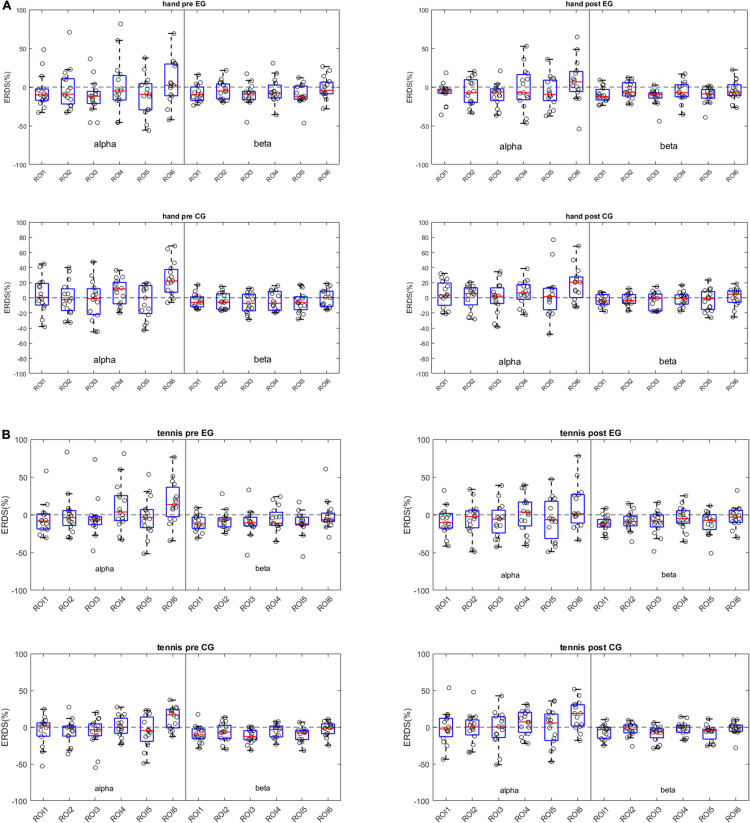
**(A)** Distribution of ERD/S patterns across frequency bands and ROIs for the hand condition. Each dot represents a subject-specific average ERD/S in the time period of the task (0.5 to 3.5 s w.r.t. the task cue) for a particular frequency band and ROI. The subplots on the left column show the ERD/S distribution for the pre-intervention period for the experimental group (top) and for the control group (bottom). The right column shows the post-intervention ERD/S distribution. **(B)** Distribution of ERD/S patterns across frequency bands and ROIs for the tennis condition. Each dot represents a subject-specific average ERD/S in the time period of the task (0.5 to 3.5 s w.r.t. the task cue) for a particular frequency band and ROI. The subplots on the left column show the ERDS distribution for the pre-intervention period for the experimental group (top) and for the control group (bottom). The right column shows the post-intervention ERD/S distribution.

In Figures 3A,B, we use boxplots to visualize summary statistics at the group level, based on ERD/S values for each condition (hand and tennis), respectively, for alpha and beta frequency bands and for all six ROIs. For both conditions, the distribution of ERD/S values shows larger variability in the alpha band than in the beta band throughout the ROIs. We can also observe that in the EG, some subjects contribute as outliers to the larger variability observed in the alpha band in the pre-intervention period (large positive ERD/S values in the top left subplot in Figures 3A,B).

Moreover, the median values among ROIs for the hand condition are more negative for the EGs than for the control group in the pre-intervention period in the alpha band. For the tennis conditions, the medians of the ROIs are similar between groups and between time periods.

### Distance Matrices and Variability Results

**FIGURE 4:**
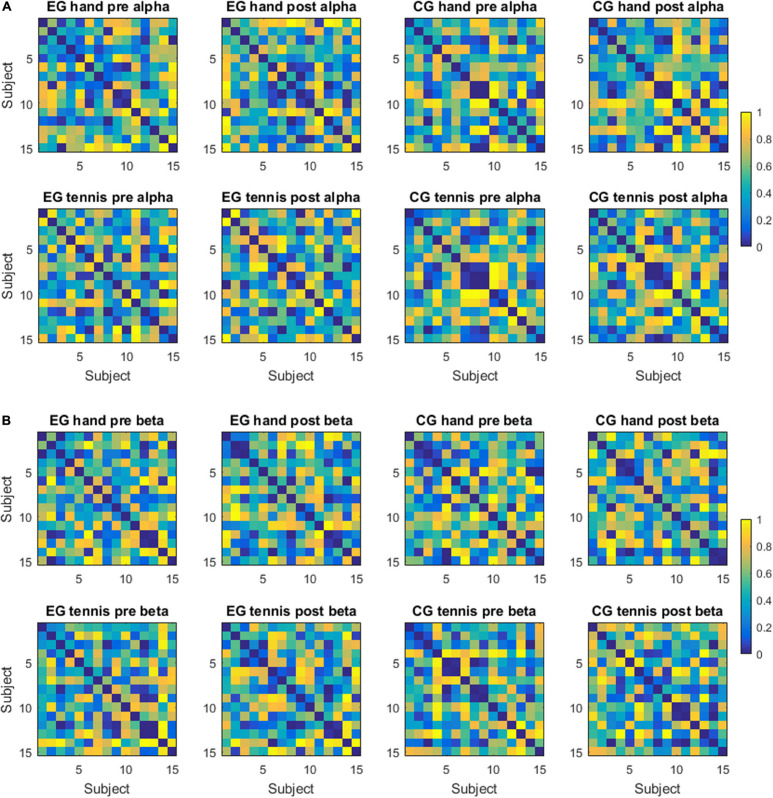
**(A)** Distance matrices—alpha frequency band. Each matrix displays the distance between subject-specific ERDS patterns in the alpha frequency band for a group (experimental or control), a condition (hand or tennis motor imagery), and a time period (pre- or post-intervention). The distance is computed as 1 - rho, where rho is the Spearman correlation. **(B)** Distance matrices—beta frequency band. Each matrix displays the distance between subject-specific ERDS patterns in the beta frequency band for a group (experimental or control), a condition (hand or tennis motor imagery) and a time period (pre- or post-intervention). The distance is computed as 1 - rho, where rho is the Spearman correlation.

Inter-individual differences are illustrated in terms of distance measures between subject-specific ERD/S patterns for the alpha band in Figure 4A and for the beta band in Figure 4B, for the factors group (EG/CG), time period (pre/post), and condition (hand/tennis). The distances are calculated between pairs of subjects considering as a pattern the ERD/S during the task period of all the channels, without averaging them. In Figure 4A, we observe some clustering in the ranking of the subjects, therefore showing smaller distances than others. Moreover, we observed a consistency in ranking across time periods both for the EG and for the control group. For the latter, we also observed similar rankings across conditions. For example, subjects 6, 8, 9, and 10 from the EG show a strong similarity, therefore small distances among each other across conditions; whereas subjects 4, 8, 9, and 12 from the control group are consistently different independent of the condition or time period, by showing large distances among each other.

In the beta band, the ranking of the subjects was more similar for the EG across conditions and time periods than for the subjects of the control group. For example, subjects 3, 8, 9, 12, and 13 were very similar in ranking across conditions and time periods. We have not observed such a consistency in the control group.

**FIGURE 5:**
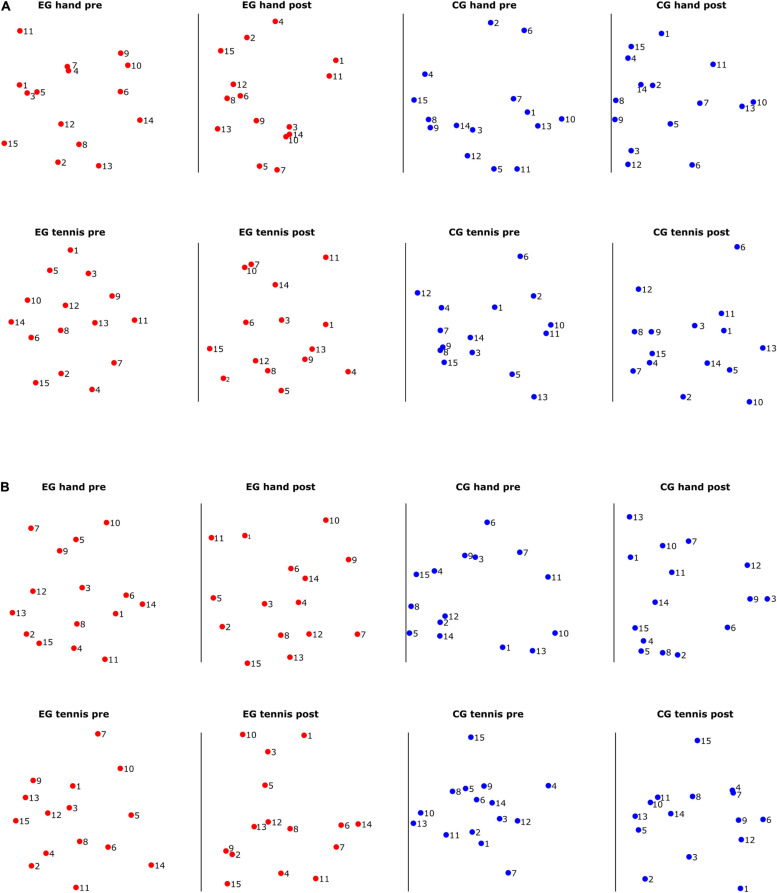
**(A)** Multidimensional scaling (MDS) scatter plots showing the relation among the subject-specific ERD/S patterns in the alpha frequency band for subjects in the experimental group (red dots) and subjects in the control group (blue dots). **(B)** Multidimensional scaling scatter plots showing the relation among the subject-specific ERD/S patterns in the beta frequency band for subjects in the experimental group (red dots) and subjects in the control group (blue dots).

For an intuitive visualization of the relationship between the ERD/S magnitudes of different subjects, we used MDS. In Figures 5A,B, we show their relation for each of the two frequency bands, respectively. With red dots we show the subjects from the EG and with blue the subjects from the control group. The closer the two dots are to one another, the more similar the magnitude of the ERD/S for the two subjects. For example, in Figure 5A, in the EG hand pre-condition, subject 1 is similar to subjects 3 and 5 but very different to subject 2 or 13. In each figure, we visualize separately the structure of the rankings of the subjects for different conditions and time periods. We observe that some subjects remain consistent in their ranking with respect to others across conditions and time periods. For example, in the EG, subjects 8, 12, and 13 maintain their similarity across conditions and time periods. Another example is subject 6 from the control group which shows a larger dissimilarity to the other subjects from the same group independent of the condition or time period.

In Figure 5B for the beta band, we also observed that some subjects cluster together, for example, for the EG, subjects 12 and 13 are close in their rankings both across conditions and time periods, and also across frequency bands, as we have seen in Figure 5A. For the control group, we observed a different ranking of the subjects between conditions and time periods compared with the consistency in ranking found in the alpha band.

**FIGURE 6:**
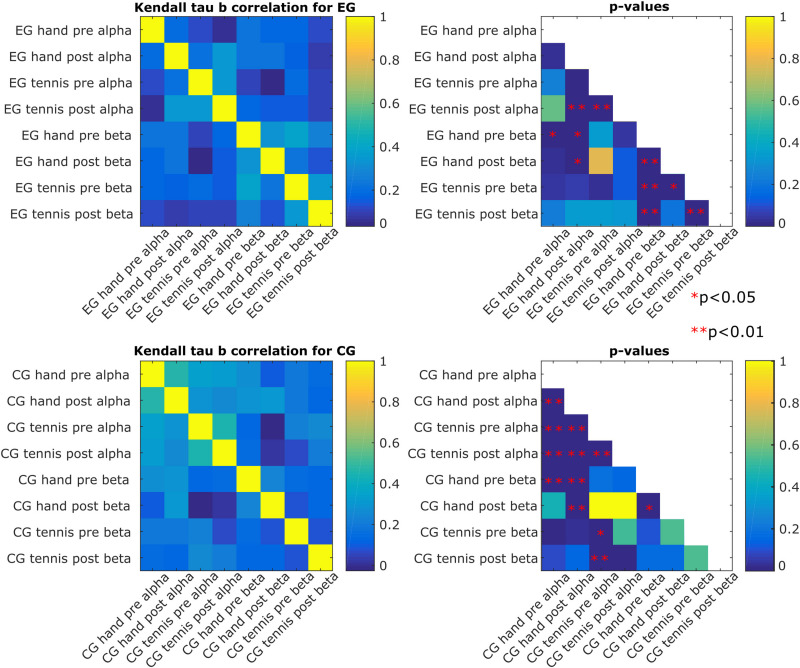
Kendall’s Tau-b correlation among the eight conditions for each of the two groups of subjects (experimental and control) and their associated *p*-values. A significant *p*-value leads to the rejection of the null hypothesis that the correlation would be 0 (i.e., independent conditions), and it indicates that the pair of conditions shares a similar distribution.

Figure 6 shows the degree of similarity between the pairs of conditions and time points in which we evaluated the rankings of the subjects. We chose Kendall’s Tau-b correlation coefficient to adjust for ties in the ranking, and we also report the associated *p*-values corrected for multiple comparisons using the Bonferroni–Holm correction. We found that the ranking of the magnitude of the ERD/S patterns for the subjects in the EG hand pre-beta was correlated with the one in the EG hand pre-alpha (τ_*b*_ = 0.23, *p* = 0.03), which indicates that the ranking of the subject is maintained across frequency bands for the hand condition in the pre-intervention time period. We also found stronger correlations between the EG tennis post-alpha and EG hand post-alpha (τ_*b*_ = 0.34, *p* = 0.0001) as well as EG tennis pre-alpha (τ_*b*_ = 0.35, *p* = 0.00009), which suggest consistency in ranking in the alpha band across conditions (τ_*b*_ = 0.32, *p* = 0.0005) and time periods. For the beta band, we observed a stronger correlation across both conditions: τ_*b*_ = 0.38, *p* = 0.000007 for tennis pre-hand pre, and time periods: τ_*b*_ = 0.32, *p* = 0.0006 for hand pre–hand post and τ_*b*_ = 0.38, *p* = 0.000007 for tennis pre-tennis post than in the alpha band for the subjects in the EG.

For the subjects in the control group, we found a stronger consistency in ranking in the alpha band than in the beta band, especially across time periods (τ_*b*_ = 0.47, *p* = 9.2^∗^e–11 for the hand pre alpha to hand post-alpha and τ_*b*_ = 0.45, *p* = 9.6^∗^e–10 for the tennis pre alpha to tennis post-alpha). The consistency across conditions was also significant: τ_*b*_ = 0.33, *p* = 0.0005 for tennis pre alpha to hand pre alpha and τ_*b*_ = 0.25, *p* = 0.006 for tennis post-alpha to hand post-alpha. In the beta band, the only significant correlation was between hand post and the hand pre (τ_*b*_ = 0.23, *p* = 0.02). The other significant correlations were found across frequency bands but for the same condition or time period (τ_*b*_ = 0.27, *p* = 0.002 for hand pre beta to hand pre alpha, τ_*b*_ = 0.29, *p* = 0.0005 for hand pre beta to hand post-alpha, and τ_*b*_ = 0.3, *p* = 0.0004 for hand post-beta to hand post-alpha). For the tennis condition, the significant correlations were τ_*b*_ = 0.22, *p* = 0.047 for pre beta to pre alpha and τ_*b*_ = 0.25, *p* = 0.009 for post-beta to pre alpha.

## Discussion

The aim of this work was to re-evaluate data from a previous study focusing on intra- and inter-individual differences in the observed brain patterns of the individuals. More concretely, we investigated ERD/S patterns during sports motor imagery and discovered high variability among the subjects. By taking into account the ERD/S patterns at the level of all the six ROIs, we assessed the dissimilarity between these patterns by means of distances. The subject-specific ERD/S values for each ROI (Figure 2) shows that some subjects elicit similar ERD/S values throughout the conditions and time points. However, we found very different ERD/S values among subjects in the EG. For example, subject EG3 shows a strong negative ERD/S value at the level of ROI3 and ROI5 in both hand and tennis pre conditions for both alpha and beta frequencies. These values slightly increase but remain negative for all the conditions and frequencies in the post-intervention period. Contrarily, another subject (EG14) presents positive ERD/S values over all conditions. Similar observations have been found in the control group, for example, subject CG3 compared with subject CG12. Moreover, we observed that the ERD/S values for the beta frequency show less variability within and among the subjects for either group compared with the alpha frequency band which was also observed in the study by Haegens et al. (2014). This variability was further assessed in the ERD/S values at the group level. For both conditions, hand and tennis, the distribution of ERD/S values shows larger variability in the alpha band than in the beta band throughout the ROIs (Figures 3A,B). Moreover, we observed that in the EG, some subjects show large positive ERD/S values in the alpha band in the pre-intervention period indicating a strong variability among this sample of participants. Especially for MI of tennis, the ERD/S patterns before the intervention phase show a high distribution across frequency bands and ROIs (Figure 3B). Based on these results, it is somehow speculative to attribute any activity changes particularly to the intervention. Beside some outliers, the variability in the beta band is quite low for all conditions and groups.

The results of inter-individual differences in terms of distance measures between subject-specific ERD/S patterns show again more differences for the alpha compared with the beta band. In the alpha band (Figure 4A), we observed some clustering in the ranking of the subjects, therefore showing smaller distances than others. Furthermore, a consistency in ranking across time periods both for the EG and for the control group exists. For the control group, we also observed similar rankings across conditions. In the beta band (Figure 4B), the ranking of the subjects was more similar for the EG across conditions and time periods than for the subjects of the control group. Moreover, the range of the variability was larger for the alpha band than for the beta band. In other words, when assessing the distance between a pair of subjects in terms of the ERD/S values in the alpha band, we can find subjects that show strong (dis)similarities with others, whereas in the beta band the magnitude of these (dis)similarities is more contained. A better visualization of the relationship between the ERD/S magnitudes of different subjects is illustrated in the MDS plots (Figures 5A,B). In the alpha band, we observe that some subjects remain consistent in their ranking with respect to others across conditions and time periods. For example, in the EG subjects 8, 12, and 13 maintain their similarity across conditions and time periods. For the beta band, we also observed that some subjects cluster together, for example, for the EG, subjects 12 and 13 are close in their rankings both across conditions and time periods, and also across frequency bands, as we have seen in Figure 5A. For the control group, we observed a different ranking of the subjects between conditions and time periods compared with the consistency in ranking found in the alpha band (Figure 6).

Haegens et al. (2014) reported similar findings of inter-subject variability in posterior alpha peak frequency by means of magnetoencephalography. They investigated how alpha peak frequency differed across cognitive conditions and ROIs within and between subjects with an N-back paradigm. Compared with beta peak frequencies, the alpha peak frequency in posterior regions increases with increasing cognitive demands and engagement. Furthermore, they showed that it is also valid across a wider frequency range than the commonly used 8–12 Hz band. This should be taken into account when comparing power values between different conditions. Moreover, they claimed that using a fixed alpha band might bias results against certain subjects and conditions. Even though many researchers observed that individual differences in brain oscillations predict certain cognitive performance (Klimesch et al., 1990a; Park et al., 2014; Jiang et al., 2015), further research considering individual oscillatory (dis)similarity is essential for a better understanding of its correlation. The variability in alpha power plays also an important role in studies investigating the resting state, especially in fMRI experiments (Laufs et al., 2003; Moosmann et al., 2003; Gonçalves et al., 2006). For example, Gonçalves et al. (2006) performed a simultaneous recording of EEG-fMRI to identify blood oxygenation level–dependent changes associated with spontaneous variations of the alpha rhythm, which is an indicator of the brain resting state (Goldman et al., 2002). Their analysis was focused on inter-subject variability associated with the resting state. Results suggest that the resting state varies over subjects and, sometimes, even within one subject. Following this, they suggested that the inter-subject variability of the resting state should be addressed when comparing fMRI results from different subjects. Although there is evidence that brain network structure differs between persons (Chu et al., 2012; Cox et al., 2018), the contribution of different frequency bands and oscillatory activity is still unknown and needs further fine-grained characterization.

Another study revealed anatomical structure of the premotor-parietal network to be an effective factor contributing to inter-individual differences in brain activation (Kasahara et al., 2015). They found that MI related patterns are associated with development of non-primary somatosensory and motor areas. In their study, they found that gray matter volume in motor-related cortical areas like the supplementary motor area (SMA) and the dorsal premotor cortex (PMd) correlated with BCI success rate. These areas are well-known as the substrates of motor imagery and planning (Hanakawa et al., 2003, 2008). Participants with greater gray matter volume in the SMA, SSA, and pre-PMd are more likely to show the desired brain activity during motor imagery to increase BCI performance. Advancing our understanding of BCI performance in relation to its neuroanatomical correlates may lead to better customization of BCIs based on individual brain structure.

Finally, the outcome of this variability analysis brings us to the following suggestions for future studies: especially in the application of motor imagery paradigms for EEG-based BCI systems, a user-centered measurement design might be beneficial. Beside the investigation of subject-specific motor-related oscillations (ERD or ERS), demographic and individual features of the participants might be relevant. For example, like we observed in our study (Wriessnegger et al., 2018), participants which are used to playing tennis frequently show different ERD/S patterns in the alpha band compared with participants being less sportive. Previous studies already reported different factors influencing BCI performance (Blankertz et al., 2010; Kübler et al., 2011; Jeunet et al., 2016); nevertheless, attention should also be paid to special sports, skills, or habits that the participant might have. Moreover, a pre-investigation of the subject-specific patterns during a certain training or intervention might be important for every future study focusing on neural correlates of motor imagery, especially when comparing experts and novices in a special cognitive task or sports performance. Generally, more attention should be paid to the composition of the sample of participants and a standard analysis of variability should always be included in the usual mean value analysis. In any case, the calculation of average parameters alone might lead to an over- or underestimation of the suspected neuronal activation patterns during motor imagery performances.

Because ERD measures are conventionally analyzed within fixed frequency bands, inter-individual differences like those we have observed in our study often occur. This means that an inter-individual difference of about 1–2 Hz is quite a common case (Klimesch, 1997). These inter-individual differences in the alpha band are primarily due to differences in memory performance (Klimesch et al., 1990b, 1993). By calculating ERD in the alpha band (Pfurtscheller and Aranibar, 1977) significant parts of alpha power will fall outside of a fixed frequency window and elicited large inter-individual variability.

To solve this problem, one can adjust the frequency bands to the individual alpha frequency (IAF) for each participant and calculate the bandwidth for the alpha frequency as a percentage of IAF (Doppelmayr et al., 1998; Goljahani et al., 2012; Grandy et al., 2013).

## Conclusion

Many authors often reported observing high inter- and intra-individual differences in brain activity among subjects but without paying much attention to it. This fact and the observation of great variability in the data of our previous study led us to perform additional (dis)similarities analysis. By calculating different distribution measurements of distances, we confirmed a high variability among participants during motor imagery primarily in the alpha frequency band. More concretely, when assessing the distance between a pair of subjects in terms of the ERD/S values in the alpha band, some subjects show strong (dis)similarities with others, whereas in the beta band the magnitude of these (dis)similarities is more contained. Moreover, we can observe that for some subjects, the within-subject ERD/S values remain similar throughout the conditions and time points; however, among subjects these values are very different. Although we identified a high variability among subjects during MI, the extent to which these inter-individual differences are a reliable indicator of the heterogeneity of a group needs to be further assessed in a longitudinal study involving further participants. In conclusion, we believe that metrics of intra- and inter-individual differences should be more frequently reported in BCI studies. These metrics could inform the development of generic BCI systems that target the adaptation among multiple users and sessions.

## Data Availability Statement

The data analyzed in this study is subject to restrictions. Requests to access these datasets should be directed to the corresponding author: s.wriessnegger@tugraz.at.

## Ethics Statement

The studies involving human participants were reviewed and approved by Medical University Graz. The patients/participants provided their written informed consent to participate in this study.

## Author Contributions

SW conducted the study, analyzed the original data, and wrote the text of the article. CB supervised the study, performed some data analysis, and proofread the article. GM-P provided the facilities for conducting the study and proofread the article. AS performed the data analysis and wrote some text of the article. All authors read and approved the final article.

## Conflict of Interest

The authors declare that the research was conducted in the absence of any commercial or financial relationships that could be construed as a potential conflict of interest.
